# Appropriate methods of evaluating future liver remnant volume to predict postoperative liver failure after major hepatectomy based on the body mass of patients with normal hepatic reserve

**DOI:** 10.1007/s00595-025-03030-0

**Published:** 2025-03-27

**Authors:** Tomohiko Ikehara, Akira Shimizu, Koji Kubota, Tsuyoshi Notake, Noriyuki Kitagawa, Hitoshi Masuo, Takahiro Yoshizawa, Kiyotaka Hosoda, Hiroki Sakai, Yuji Soejima

**Affiliations:** Division of Gastroenterological, Hepato-Biliary-Pancreatic, Transplantation and Pediatric Surgery, Department of Surgery, Shinshu University School of Medicine, Asahi 3-1-1, Matsumoto, Nagano 390-8621 Japan

**Keywords:** Body mass, Body mass index, Future liver remnant volume, Major hepatectomy, Post-hepatectomy liver failure

## Abstract

**Purpose:**

Several parameters are used to assess future liver remnant (FLR) size before major hepatectomy. This study aimed to clarify which is the most appropriate method to use for the prediction of post-hepatectomy liver failure (PHLF).

**Methods:**

The subjects of this study were 307 patients with Child–Pugh class A only, who underwent major hepatectomy, to focus on FLR size. The parameters we evaluated for their accuracy in predicting Grade B/C PHLF (PHLF B/C) using receiver operating characteristic curve analysis were FLR volume (FLRV), the FLRV to total liver volume ratio (FLRV/TLV), standard liver volume (FLRV/SLV), and body weight (FLRV/BW) according to body mass.

**Results:**

The predictive value accuracy of these four parameters for PHLF was similar for the entire cohort. However, in the subgroup analysis based on body mass index, FLRV/BW accuracy was highest in the obese group, whereas that of FLRV/TLV was highest in the lean group. Multivariate analysis identified that FLRV/BW (< 0.7%) and blood loss (≥ 1000 ml) were independent risk factors for PHLF B/C in the obese group. In the lean group, FLRV/TLV (< 40%) and biliary reconstruction were risk factors for PHLF B/C.

**Conclusions:**

The FLR size evaluation method for predicting PHLF should be appropriately selected based on the patient’s body mass.

**Supplementary Information:**

The online version contains supplementary material available at 10.1007/s00595-025-03030-0.

## Introduction

Hepatectomy is being performed increasingly for patients with malignant or benign diseases and living donors worldwide [1, 2]. The short- and long-term outcomes after hepatectomy have improved with advances in imaging systems, surgical techniques, perioperative management, and a better understanding of the liver anatomy [3]. However, post-hepatectomy liver failure (PHLF) occurs in 3.8–34% of patients and remains a potentially life-threatening complication directly associated with the procedure [4, 5].

To prevent PHLF, the patient’s liver function must be assessed preoperatively. Generally, the Child–Pugh classification, the presence or absence of portal hypertension, and the ALBI grade are used as methods to assess preoperative liver function. In Asia, the indocyanine green (ICG) test is also used commonly to assess preoperative liver function and the results of this test help define the safe extent of liver resection [6]. However, although the ICG test is very useful, it is not used widely outside Asia.

In addition to the assessment of liver function, the future liver remnant volume (FLRV) is an important factor in the prevention of PHLF. Other methods of assessing FLRV have been reported in addition to the ratio to total liver volume (FLRV/TLV), which uses the ratio to body weight (FLRV/BW) and the ratio to standard liver volume (SLV) calculated from body surface area (BSA) (FLRV/SLV) [7–9]. However, it remains unclear which method of assessment is the most valid [10].

It is generally accepted that the size of the liver is determined by the individual patient’s body mass, and many methods of calculating the SLV are based on body mass [11]. However, these calculation formulae are based on patients with a standard body mass and may not be useful in non-standard cases. We hypothesized that the appropriate method for assessing FLRV may vary according to differences in body mass. In the present study, we compared methods of assessing FLRV for different body masses based on the BMI of patients with normal hepatic reserve only, to identify the best method of assessing FLRV.

## Methods

**Fig. 1:**
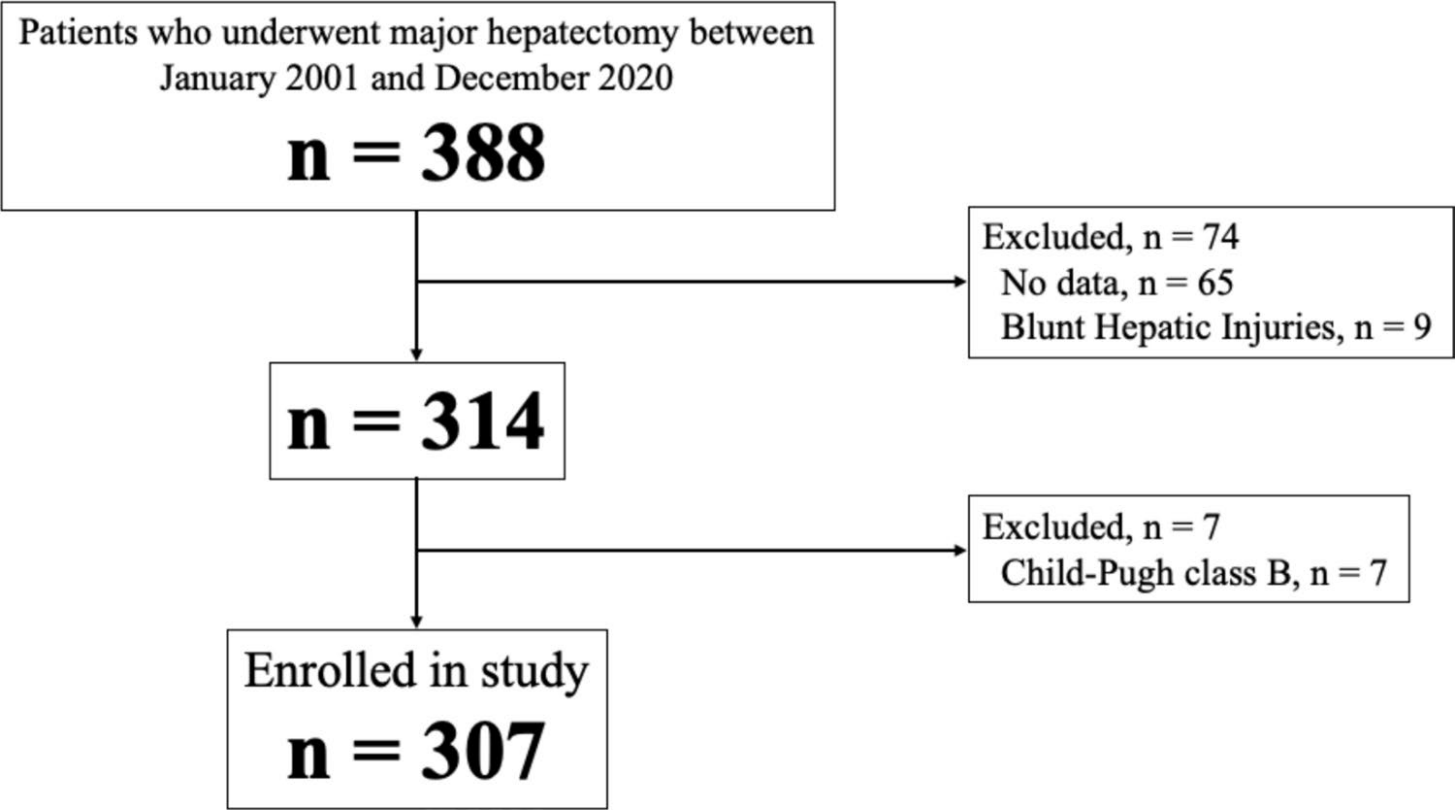
Study flow diagram

This study included 388 patients who underwent major hepatectomy at Shinshu University Hospital between January, 2001 and December, 2020. Major hepatectomy was defined as resection involving three or more Couinaud segments. After the exclusion of 65 patients with no clinical information, 9 who underwent an emergency operation for blunt hepatic injuries where the preoperative liver volume could not be calculated, and 7 with Child–Pugh class B, 307 patients were the subjects of this retrospective analysis (Fig. 1). The study was approved by the Shinshu University Hospital Ethics Committee on 17 January, 2024 (approval no. 5729) and it conformed to the provisions of the Declaration of Helsinki. Informed consent was obtained from an opt-out website and patients who declined to participate were excluded.

### Perioperative management

The targeted diseases included hepatocellular carcinoma, perihilar cholangiocarcinoma, intraductal papillary neoplasm of the bile duct, gallbladder cancer, metastatic liver tumors, and other benign liver diseases. Patients with clinical jaundice underwent preoperative biliary drainage (PBD) using endoscopic retrograde or percutaneous transhepatic biliary drainage. PBD is typically performed unilaterally on the future remnant hemiliver. Bile juice was collected using a drainage catheter and administered orally until surgery. An ICG test and general blood sampling were routinely performed preoperatively. The FLRV was calculated using Organ Volume Analysis (Hitachi Medical Corporation, Chiba, Japan) for patients up until the end of August, 2019 and then using Synapse Vincent FN-7941 (Fujifilm, Tokyo, Japan) for patients from September, 2019. The indications for hepatectomy were determined according to Makuuchi’s criteria [6] and ICG clearance of the liver remnant [12]. Major hepatectomy with resection of the caudate lobe and the extrahepatic bile duct was performed routinely for perihilar cholangiocarcinoma. Hematological examination and intra-abdominal fluid collection using ultrasonography were performed daily during the first week after hepatectomy.

### Definitions and endpoint assessment

PHLF was defined according to the criteria proposed by the International Study Group of Liver Surgery (ISGLS), namely, an elevated international normalized ratio (INR) and concurrent hyperbilirubinemia on postoperative day 5 [4]. The institution INR cut-off values for prothrombin time and serum bilirubin concentration were 1.15 and 1.40 mg/dl, respectively. Patients with PHLF were further classified into three groups according to the severity of liver failure. Grade A PHLF was defined as postoperative liver dysfunction that required no change in clinical management. Grade B PHLF was defined as deviation from the regular course that did not require invasive therapy. Grade C PHLF was based on the need for invasive treatments, such as hemodialysis, intubation and ventilation, extracorporeal liver activation, rescue hepatectomy, or transplantation.

Patient demographics and perioperative factors, including liver volume, postoperative outcomes, postoperative complications, and survival, were collected retrospectively from medical records. Postoperative complications were categorized based on the Clavien–Dindo (CD) classification [13].

The following parameters were evaluated for their accuracy in predicting Grade B/C PHLF (PHLF B/C) using receiver operating characteristic (ROC) curve analysis: FLRV, FLRV/TLV, FLRV/SLV, and FLRV/BW. The SLV calculation method was based on Urata’s formula [11]. Subsequently, a subgroup analysis was performed to examine the appropriate evaluation method according to the patient’s body mass. For a subgroup analysis, we divided the patients into three groups according to body mass using body mass index (BMI) quartiles. Patients in the first and third quartiles were classified as the standard group (Group S), those above this range were classified as the obese group (Group O), and those below this range were classified as the lean group (Group L).

### Statistical analysis

Results are presented as median values and ranges. Categorical variables were analyzed using the *χ*^2^ or Fisher’s exact test and quantitative variables were analyzed using the Wilcoxon rank-sum test. Quantitative variables were categorized into two groups according to the cut-off value obtained from the ROC curve. To identify significant independent predictive factors for PHLF B/C, we performed multivariate analysis using logistic regression analysis after covariate selection using a forward stepwise method to determine which variables to include, considering the number of outcomes in this study and eliminating potential confounders. For the multivariate analysis, we targeted covariates that were significant (*p* < 0.05) in univariate analysis. Odds ratios (ORs) and 95% confidence intervals (CIs) were calculated for the incidence of PHLF B/C. All statistical analyses were performed using JMP Pro 16.2 (SAS Institute, Inc., Cary, NC, USA). Significance was set at *p* < 0.05.

## Results

### Patient characteristics and surgical outcomes

The median BMI was 21.7 (14.7–31.7) and the first and third quartiles of BMI were 19.8 and 23.4, respectively. For preoperative management, portal vein embolization (PVE) was performed in 82 (27%) patients and biliary drainage was performed in 132 (43%) patients. The median FLRV, FLRV/TLV, FLRV/SLV, and FLRV/BW ratios were 553 (134–1242) ml, 51.9% (28.4–94.0%), 48.8% (25.2–107.8%), and 0.987% (0.464–2.034%), respectively. Pancreatoduodenectomy (PD), vascular reconstruction, and biliary reconstruction were performed simultaneously with hepatectomy in 26 (8%), 47 (15%), and 171 (56%) patients, respectively. The median operative time was 613 (238–1330) min, with a median blood loss of 570 (95–6600) ml. Blood transfusions were administered to 115 patients (37%) during surgery.

PHLF B/C developed in 43 (14.0%) of the 307 enrolled patients, 3 of whom had grade C PHLF. The frequency of PHLF B/C was similar in each subgroup, according to the BMI (Group S: *n* = 22 (14.2%), Group O: *n* = 10 (13.2%), Group L: *n* = 11 (14.5%), Group S vs. O, *p* = 0.831; Group S vs. L, *p* = 0.954; Group O vs. L, *p* = 0.814). The morbidity rate of CD grade $\ge$ IIIa, excluding PHLF, was 33.2% (*n* = 102) and the 90-day mortality rate was 1% (*n* = 3). Among the preoperative factors, sex ratio (*p* = 0.045), albumin level (*p* = 0.008), PBD (*p* < 0.001), and PVE (*p* < 0.001) were significantly different between the patients with and those without PHLF B/C (Table 1). In this cohort, more than 80% of patients had normal liver function with ICG-R15 < 15%, and there was no significant difference in ICGK or ICG-R15 < 15% between patients with and those without PHLF B/C. All parameters related to remnant liver volumes, such as FLRV, FLRV/TLV, FLRV/SLV, and FLRV/BW, were significantly lower in patients with PHLF B/C than in those without PHLF B/C (*p* = 0.004, < 0.001, < 0.001, and < 0.001, respectively). Among the perioperative factors, PD (*p* = 0.010), biliary reconstruction (*p* < 0.001), operative time (*p* < 0.001), blood loss (*p* < 0.001), and blood transfusion (*p* < 0.001) were also significantly different between patients with and those without PHLF B/C (Table 2).

**Table 1:** Background characteristics of the study population according to the development of post-hepatectomy liver failure

Parameters	Overall (*n* = 307)	Grade B or C PHLF		*p*
		Yes (*n* = 43)	No (*n* = 264)	
Background data				
Age, years^a^	69 (1–88)	70 (48–83)	69 (1–88)	0.191
Sex ratio (M: F)	186:121	32:11	154:110	0.045
BMI, kg/m^2a^	21.7 (14.7–31.7)	21.6 (15.6–31.7)	21.7 (14.7–31.7)	0.969
19.8 ≤ BMI ≤ 23.4	155 (50%)	22 (51%)	133 (50%)	
BMI > 23.4	76 (25%)	10 (23%)	66 (25%)	
BMI < 19.8	76 (25%)	11 (26%)	65 (25%)	
ASA score				0.487
1	104 (38%)	14 (35%)	90 (38%)	
2	155 (56%)	25 (63%)	130 (55%)	
3	17 (6%)	1 (3%)	16 (7%)	
Comorbidity				
Diabetes mellitus	50 (16%)	9 (21%)	41 (16%)	0.374
Chronic hepatitis / Hepatic cirrhosis	49 / 7 (16% / 2%)	8 / 0 (19% / 0%)	41 / 7 (16% / 3%)	0.947
Preoperative cholangitis	50 (16%)	10 (23%)	40 (15%)	0.182
Preoperative management				
Preoperative biliary drainage				< 0.001
Yes	132 (43%)	32 (74%)	100 (38%)	
No	175 (57%)	11 (26%)	164 (62%)	
Portal vein embolization				< 0.001
Yes	82 (27%)	25 (58%)	57 (22%)	
No	225 (73%)	18 (42%)	207 (78%)	
Preoperative blood tests^a^				
Total bilirubin, mg/dl^a^	0.73 (0.06–2.71)	0.76 (0.34–2.71)	0.73 (0.06–2.27)	0.668
AST, units/l^a^	30 (13–129)	37 (18–74)	30 (13–129)	0.612
ALT, units/l^a^	30 (9–229)	37 (16–93)	28 (9–229)	0.864
Albumin, g/dl^a^	3.8 (2.8–4.7)	3.6 (2.8–4.3)	3.8 (2.8–4.7)	0.008
Prothrombin time, %^a^	96 (67–135)	94 (73–126)	97 (67–135)	0.729
Platelet count, × 10^4^/μl^a^	20.3 (7.2–43.4)	18.9 (9.9–27.9)	20.8 (7.2–43.4)	0.074
mGPS				0.485
0	124 (87%)	28 (93%)	96 (85%)	
1	10 (7%)	1 (3%)	9 (8%)	
2	9 (6%)	1 (3%)	8 (7%)	
ICGK^a^	0.16 (0.10–0.36)	0.16 (0.10–0.28)	0.16 (0.11–0.36)	0.192
ICG-R15 < 15%	240 (82%)	32 (76%)	208 (83%)	0.279
Parameters on remnant liver volume				
FLRV, ml^a^	553 (134–1241)	415 (299–1074)	572 (134–1241)	0.004
FLRV/TLV, %^a^	51.9 (28.4–94.0)	41.5 (31.0–88.6)	53.9 (28.4–94.0)	< 0.001
FLRV/SLV, %^a^	48.8 (25.2–107.8)	38.3 (27.6–88.2)	52.0 (25.2–107.8)	< 0.001
FLRV/BW, %^a^	0.987 (0.464–2.034)	0.778 (0.519–2.034)	1.013 (0.464–2.013)	< 0.001

*PHLF* post-hepatectomy liver failure, *ASA–PS* American Society of Anesthesiologists physical status, *AST* aspartate aminotransferase, *ALT* alanine aminotransferase, *mGPS* modified Glasgow Prognostic Score, *ICGK* plasma clearance rate of indocyanine green (ICG), *ICG-R15* indocyanine green retention at 15 min, *FLRV* future liver remnant volume, *TLV* total liver volume, *SLV* standard liver volume, *BW* body weight

^a^values are expressed as the median (range)

**Table 2:** Surgical and postoperative data of the study population according to the development of post-hepatectomy liver failure

Parameters	Overall (*n* = 307)	Grade B or C PHLF		*p*
		Yes (*n* = 43)	No (*n* = 264)	
Type of hepatectomy				0.002
Right hepatectomy	164 (53%)	32 (74%)	132 (50%)	
Left hepatectomy	115 (37%)	5 (12%)	110 (42%)	
Right trisectionectomy	9 (3%)	1 (2%)	8 (3%)	
Left trisectionectomy	12 (4%)	4 (9%)	8 (3%)	
Central hepatectomy	7 (2%)	1 (2%)	6 (2%)	
Vascular reconstruction				0.790
Yes	47 (15%)	6 (14%)	41 (16%)	
No	260 (85%)	37 (86%)	223 (84%)	
Pancreatoduodenectomy (PD)				0.010
Yes	26 (8%)	8 (19%)	18 (7%)	
No	281 (92%)	35 (81%)	246 (93%)	
Other organ resection				0.087
Yes	17 (6%)	0 (0%)	17 (6%)	
No	290 (94%)	43 (100%)	247 (94%)	
Biliary reconstruction				< 0.001
Yes	171 (56%)	36 (84%)	135 (51%)	
No	136 (44%)	7 (12%)	129 (49%)	
Operative time, min^a^	613 (238–1330)	694 (272–1297)	598 (238–1330)	< 0.001
Blood loss, ml^a^	570 (95–6600)	1000 (130–6600)	550 (95–6600)	< 0.001
Blood transfusion				< 0.001
Yes	115 (37%)	27 (63%)	88 (33%)	
No	192 (63%)	16 (37%)	176 (67%)	
Total clamping time, min^a^	60 (0–210)	60 (0–210)	60 (0–150)	0.230
Morbidity excluding PHLF				
All grade	219 (71%)	35 (81%)	183 (69%)	0.106
Clavien–Dindo classification grade ≥ IIIa	102 (33%)	24 (56%)	78 (30%)	< 0.001
Grade B or C post-hepatectomy bile leakage	35 (11%)	10 (23%)	25 (9%)	0.008
90-day mortality, *n* (%)	3 (1%)	3 (7%)	0 (0%)	< 0.001
Postoperative hospital stays, day^a^	26 (8–287)	48 (14–209)	23 (8–287)	< 0.001

PHBL was defined according to the criteria proposed by the International Study Group of Liver Surgery (ISGLS)

*PHLF* post-hepatectomy liver failure, *PD* pancreaticoduodenectomy, *CD* Clavien–Dindo

^a^Values are expressed as the median (range)

### ROC analysis of parameters on remnant liver volume for predicting PHLF B/C

**Fig. 2:**
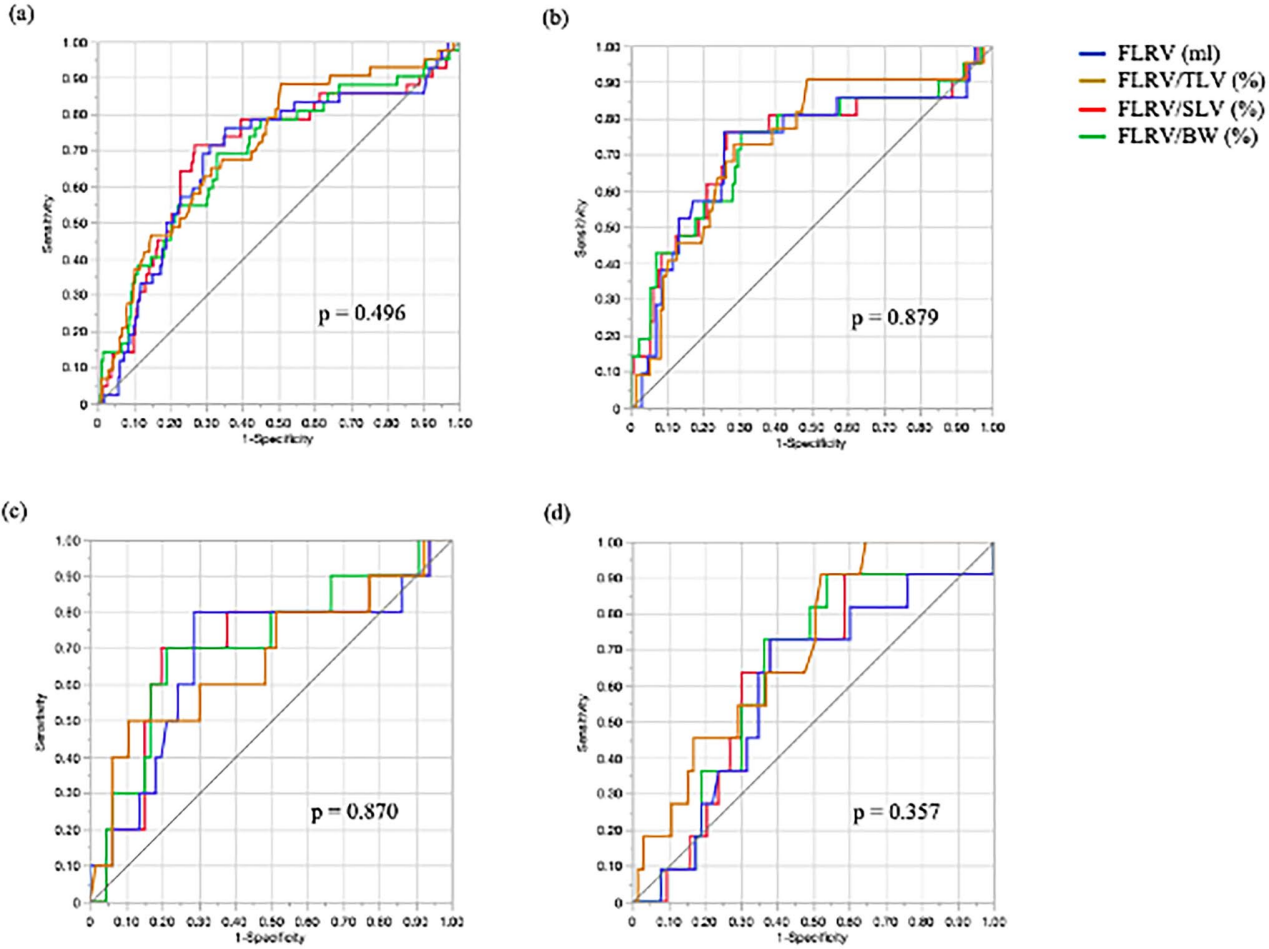
Receiver operating characteristic curve (ROC) and area under the curve (AUC) for four parameters related to remnant liver volumes. **a** Comparison of each AUC for all the patients. **b** Comparison of the AUC in the standard body mass group. **c** Comparison of the AUC in the obese group. **d** Comparison of each AUC in the lean group. Blue, ROC of FLRV; Brown, ROC of FLRV/TLV; Red, ROC of FLRV/SLV; Green, ROC of FLRV/BW. The *p*-values listed here refer to the probability of an overall hypothesis test for the null hypothesis that “all AUCs are equal”

The predictive accuracy of the four parameters for PHLF B/C, which assesses remnant liver volume, was evaluated using ROC analysis. In all patients, the area under the curve (AUC) of FLRV, FLRV/TLV, FLRV/SLV, and FLRV/BW was 0.684 (95% CI 0.584–0.769; *p* = 0.002), 0.711 (95% CI 0.620–0.788; *p* < 0.001), 0.693 (95% CI 0.592–0.779; *p* < 0.001), and 0.688 (95% CI 0.589–0.773; *p* < 0.001), respectively (Table 3). The cut-off values of each parameter for predicting PHLF B/C were 495 ml, 40%, 42%, and 0.702%, with sensitivities of 0.646, 0.852, 0.732, and 0.887, and specificities of 0.762, 0.465, 0.714, and 0.381, respectively. Although no significant differences were observed when comparing the AUC (*p* = 0.496, Fig. 2a), the AUC of FLRV/TLV was the highest among all the parameters.

**Table 3:** Area under the curve for each parameter of remnant liver volume to predict grade B or C post-hepatectomy liver failure in all the patients

Parameters	All patients (*n* = 307)	
	AUC (95% CI)	*p*
FLRV	0.684 (0.584, 0.769)	0.002
FLRV/TLV	0.711 (0.620, 0.788)	< 0.001
FLRV/SLV	0.693 (0.592, 0.779)	< 0.001
FLRV/BW	0.688 (0.589, 0.773)	< 0.001

The values in parentheses are the 95% confidence intervals (CIs). The area under the curve (AUC) for each parameter was calculated from receiver operating characteristic (ROC) curve analyses to predict grade B or C post-hepatectomy liver failure

*PHLF* post-hepatectomy liver failure, *FLRV* future liver remnant volume, *TLV* total liver volume, *SLV* standard liver volume, *BW* body weight

### Subgroup analysis according to body mass

Considering that body mass affects liver volume, we examined the predictive accuracy of the four liver volume assessment parameters mentioned above for predicting the incidence of PHLF B/C according to body mass based on BMI (Table 4). Group S (19.8 ≤ BMI ≤ 23.4) comprised 155 patients, Group O ((BMI > 23.4) comprised 76 patients, and Group L (BMI < 19.8) comprised 76 patients. In Group S, all parameters predicted PHLF (AUC: FLRV 0.720, FLRV/TLV 0.732, FLRV/SLV 0.728, and FLRV/BW 0.727) accurately, and there was no significant difference among the parameters (Table 4, Fig. 2b). Conversely, in Group O, FLRV/BW had the highest AUC (0.708, *p* = 0.056) among the four parameters (AUC: FLRV 0.680, FLRV/TLV 0.670, and FLRV/SLV 0.700), and only FLRV/BW had a p-value less than 0.06. This indicates that FLRV/BW could predict PHLF most accurately among these parameters (Table 4, Fig. 2c). The cut-off value of FLRV/BW for predicting PHLF B/C in the obese group was 0.7%, with sensitivity of 0.833 and specificity of 0.600.

**Table 4:** Area under the curve of each parameter on remnant liver volume for predicting grade B or C post-hepatectomy liver failure according to body mass

Parameters	Standard body mass with 19.8 ≤ BMI ≤ 23.4 (*n* = 155)		Obese cases with BMI > 23.4 (*n* = 76)		Lean cases with BMI < 19.8 (*n* = 76)		
	AUC (95% CI)	*p*	AUC (95% CI)	*p*	AUC (95% CI)	*p*	
FLRV	0.720 (0.566, 0.836)	0.008	0.680 (0.449, 0.847)	0.092	0.597 (0.410, 0.759)		0.394
FLRV/TLV	0.732 (0.599, 0.834)	< 0.001	0.670 (0.438, 0.841)	0.118	0.701 (0.535, 0.826)		0.020
FLRV/SLV	0.728 (0.572, 0.844)	0.003	0.700 (0.472, 0.859)	0.069	0.625 (0.442, 0.778)		0.270
FLRV/BW	0.727 (0.572, 0.841)	0.003	0.708 (0.486, 0.861)	0.056	0.636 (0.456, 0.785)		0.261

Values in parentheses are 95% confidence intervals (CIs). The area under the curve (AUC) for each parameter was calculated from receiver operating characteristic (ROC) curve analyses for predicting grade B or C post-hepatectomy liver failure

*PHLF* post-hepatectomy liver failure, *FLRV* future liver remnant volume, *TLV* total liver volume, *SLV* standard liver volume, *BW* body weight, *BMI* body mass index

In Group L, FLRV/TLV had the highest AUC (0.701, *p* = 0.020), it was the only one with a *p*-value < 0.050 and the only one that could be predicted significantly (AUC: FLRV 0.597, FLRV/SLV 0.625, and FLRV/BW 0.636) (Table 4, Fig. 2d). The cut-off value of FLRV/TLV for predicting PHLF B/C in Group L was 40%, with sensitivity of 0.831 and specificity of 0.455.

### Risk factors for PHLF B/C by body mass

Table 5 shows the results of the univariate and multivariate analyses evaluating the risk factors for developing PHLF B/C in Groups O and L. The liver volume assessment parameters and factors associated with the development of PHLF B/C, shown in Table 1 and Table 2, were used as covariates in this analysis. First, in the univariate analysis of Group O, FLRV/BW (< 0.7%) (*p* = 0.002), PD (*p* = 0.005), low serum albumin (< 3.5 g/dl) (*p* = 0.013), blood loss (> 1000 ml) (*p* = 0.009), PVE (*p* = 0.036), and preoperative cholangitis (*p* = 0.031) were associated with the development of PHLF B/C. Multivariable logistic regression analysis, with variables that showed significant differences in the univariate analysis, identified FLRV/BW below 0.7% (OR 7.98, 95% CI 1.57–404.53; *p* = 0.012) and blood loss above 1000 ml (OR 6.17, 95% CI 1.22–313.27; *p* = 0.028) as the factors associated with an increased risk of the development of PHLF B/C in Group O.

**Table 5:** Univariate and multivariate analyses of risk factors for Grade B or C post-hepatectomy liver failure

Parameters	No. of patients	Univariate			Multivariate		
		Odds ratio	95% CI	*p*	Odds ratio	95% CI	*p*
Obese patients with a BMI > 23.4 (*n* = 76)							
FLRV/BW^a^							
< 0.7%	17	7.50	1.81–31.06	0.002	7.98	1.57–40.53	0.012
≥ 0.7%	59	1.00			1.00		
Pancreatoduodenectomy							
Yes	3	16.25	1.32–200.01	0.005	5.83	0.39–86.92	0.201
No	73	1.00			1.00		
Albumin^a^							
< 3.5 g/dl	20	5.25	1.30–21.24	0.013			
≥ 3.5 g/dl	53	1.00					
Blood loss^a^							
≥ 1000 ml	20	5.57	1.38–22.50	0.009	6.17	1.22–31.27	0.028
< 1000 ml	56	1.00			1.00		
Portal vein embolization							
Yes	18	4.08	1.03–16.21	0.036			
No	58	1.00					
Preoperative cholangitis							
Yes	8	5.19	1.02–26.72	0.031			
No	68	1.00					
Lean cases with BMI < 19.8 (*n* = 76)							
FLRV/TLV^a^							
< 40%	16	4.09	1.17–18.69	0.032	4.29	1.01–18.14	0.048
≥ 40%	60	1.00			1.00		
Portal vein embolization							
Yes	22	5.83	1.50–22.66	0.006			
No	54	1.00					
Biliary reconstruction							
Yes	44	9.12	1.10–75.39	0.017	9.45	1.10–80.87	0.040
No	32	1.00			1.00		
Blood loss^a^							
≥ 550 ml	30	22.50	2.70–187.83	< 0.001			
< 550 ml	46	1.00					
Blood transfusion							
Yes	26	12.71	2.49–64.78	< 0.001			
No	50	1.00					
Operative time^a^							
≥ 620 min	33	7.69	1.53–38.57	0.006			
< 620 min	43	1.00					
Preoperative biliary drainage							
Yes	35	6.75	1.35–33.79	0.010			
No	41	1.00					

*PHLF* post-hepatectomy liver failure, *FLRV* future liver remnant volume, *TLV* total liver volume, *BW* body weight, *BMI* body mass index, *PHLF* post-hepatectomy liver failure

^a^Cut off values for continuous variable factors were calculated from ROC curves

In Group L, univariate analysis revealed that FLRV/TLV (< 40%) (*p* = 0.032), PVE (*p* = 0.006), biliary reconstruction (*p* = 0.017), blood loss (> 550 ml) (*p* < 0.001), blood transfusion (*p* < 0.001), operative time (≥ 620 min) (*p* = 0.006), and PBD (*p* = 0.010) were associated with the development of PHLF B/C. Multivariable logistic regression analysis identified that FLRV/TLV below 40% (OR 4.29, 95% CI 1.01–18.14; *p* = 0.048) and biliary reconstruction (OR 9.45, 95% CI 1.10–80.87; *p* = 0.040) were independent risk factors for PHLF B/C in Group L.

## Discussion

This study focused on the volume of the remnant liver and investigated the most appropriate method for evaluating remnant liver volume to predict liver failure after major hepatectomy according to body mass, targeting patients whose liver function was considered normal with Child–Pugh class A. Our results indicate that any FLRV assessment method can predict PHLF B/C in patients with standard body mass. However, the accuracy of the predictive value for liver failure after hepatectomy differed according to the FLRV assessment method in obese versus lean patients. FLRV/BW is more useful in obese patients, whereas FLRV/TLV is more useful than other FLRV assessment methods in lean patients. To our knowledge, this is the first study to investigate appropriate methods of evaluating FLRV for predicting PLHF after major hepatectomy according to body mass. We believe that our findings will contribute to the prevention of PHLF by improving the accuracy of PHLF prediction using an appropriate FLRV evaluation method.

The importance of FLRV for predicting postoperative liver function has been reported [14] and there are multiple methods for evaluating liver remnant volume. This study used four representative factors as parameters for liver volume evaluation: FLRV, FLRV/TLV, FLRV/SLV, and FLRV/BW. According to Guglielmi et al., the safe resection limit is 25% of the TLV for patients with normal liver function, but for patients with impaired livers and cirrhosis, biliary stasis, or fatty deposits, the limit of FLRV/TLV is 30–40%. However, the evaluation of liver function is required in addition to volume [15]. According to one report, FLRV/SLV is more specific than FLRV/TLV for predicting postoperative course after major hepatectomy: a FLRV/SLV ≤ 20% is associated with a higher risk of PHLF developing in patients with non-cirrhotic livers and a FLRV/SLV ≤ 30% is associated with a higher risk of PHLF developing in patients with cirrhosis [16]. Moreover, it has been reported that TLV decreases with the progression of cirrhosis, and that the decrease in TLV/SLV is especially marked in patients with poor hepatic function because cirrhotic livers have lower levels of hepatocyte growth factor and impaired transcription factors, leading to a reduction in DNA synthesis and lower volumes of the regenerated liver [17, 18]. Therefore, FLRV/SLV is more useful than FLRV/TLV for predicting liver function after hepatectomy in patients with cirrhosis. FLRV/BW originates from the concept of graft-to-recipient weight ratio (GRWR) at the time of liver transplantation, and a GRWR of 0.6–0.8% or higher is considered necessary for safe liver transplantation [19]. Specifically, the cut-off values of FLRV/BW for PHLF prediction have been reported as 0.55% for non-cirrhotic livers [20] and 1.4% for cirrhotic livers [21].

No reports have comprehensively compared which of the above parameters of liver volume is most useful for predicting PHLF after hepatectomy. On examining this, we discussed the possibility that liver volume requirements may differ according to body mass. Hwang et al. reported that TLV is influenced by BMI and sex, with a higher BMI indicating a larger liver [22]. Consistent with their previous report, our study also showed a positive correlation between BMI and TLV, as calculated using CT (Online Resource 1, *p* < 0.001). Conversely, Um et al. showed that BMI stratified the mean SLV [23]. This suggests that if the body mass is outside of the normal range, SLV-based assessment methods will not be useful for evaluating liver function because the SLV and actual TLV will differ. Furthermore, Truant et al. noted that FLRV/BW was superior to FLRV/SLV for identifying patients with obesity, who were at risk of PHLF [24]. These results suggest that the appropriate liver volume assessment method may differ according to body mass. The predictive accuracy of remnant liver volume assessment for PHLF development is expected to be improved by stratification according to body mass. According to our data using stratification based on BMI, liver volume assessment methods that could more accurately predict PHLF differed according to body mass. Moreover, it was confirmed that the cut-off values of each parameter were not significantly different for each subgroup (Online Resource 2).

For patients with obesity (BMI > 23.4 kg/m^2^), FLRV/BW was the most accurate predictor of PHLF development among the four FLRV evaluation methods and was identified as an independent risk factor for PHLF. Although the lack of sufficient clinical information on fatty liver made it difficult to assess the background liver histology, we attributed this result to the fact that there were more patients with fatty liver in Group O than in the other groups. As shown in Online Resource 3, Group O tended to have higher DM prevalence (Group O: *n* = 16 [21.1%], Group S: *n* = 24 [15.5%], Group L: *n* = 10 [13.2%], Group O vs. S, *p* = 0.299; Group O vs. L, *p* = 0.195), serum AST (median, Group O: 37, Group S: 28, Group L: 32, Group O vs. S, *p* = 0.107; Group O vs. L, *p* = 0.285), and ALT levels (median, Group O: 45, Group S: 26, Group L: 24, Group O vs. S, *p* = 0.133; Group O vs. L, *p* = 0.092). However, there was no significant difference from the other groups, providing weak support for the above prediction. In fatty livers, it was suspected that liver function per volume was reduced because of the presence of hepatocytes with accumulated triglycerides, a decrease in the volume of normally functioning hepatocytes, and an increase in liver volume. Therefore, the dispersion of liver function per volume between patients was also larger than that in other groups, and the evaluation method based on actual TLV might reduce the accuracy of predicting PHLF. This result is consistent with previous reports that FLRV/BW, unlike FLRV/TLV, is not based on the assumption of homogeneity of overall liver function [9] and has the advantage of reflecting the minimal metabolic requirements of the patient’s body [25], making it more useful than other FLRV evaluation methods for predicting PHLF. Moreover, although BW is generally thought to be influenced by multiple factors such as age, the effect of FLRV/BW on PHLF was generally similar even when subgroup analysis was performed by age (< 70 years vs. ≥ 70 years) (Online Resource 4). Conversely, in lean patients (< 19.8 kg/m^2^), the FLRV/TLV ratio was the most accurate predictor of PHLF development among the four FLRV evaluation methods and was identified as an independent risk factor. We considered that the reason TLV should be used as the basis for evaluating FLRV in lean patients is that the estimated liver volume calculated from body mass and weight tends to be low in lean patients.

We have additional data to support the fact that the calculated SLV may not accurately assess the liver volume required by patients in the lean and obese groups. As shown in Online Resource 2, the cut-off value of the FLRV/SLV ratio was higher in the lean patient group and lower in the obese patient group than that in the standard body mass patient group, thereby confirming the prediction that SLV was underestimated in the lean patient group and overestimated in the obese group compared with the originally required liver volume. We also noted that the FLRV/SLV ratio is useful for patients with standard body mass but inappropriate for those deviating from the norm.

In this study, we used a different BMI cut-off for body mass from the WHO classification. The reason for this is because the WHO classification would have resulted in a sample size of 42 obese patients and 33 lean patients in this cohort, which may not have been sufficient for statistical analysis. The WHO expert consultation also suggests that it may be necessary to consider setting a lower cut-off point for the obese group to identify risk factors for diabetes and cardiovascular disease, as Asian populations, including the Japanese population, have a lower BMI and higher body fat percentage than Western populations [26]. Therefore, we decided that it was not appropriate to analyze the Japanese cohort under the WHO classification, as in the present study, and a statistically valid classification method, that is, dividing body mass into BMI quartiles, was used.

This study had some limitations. First, it was a single-center retrospective study, and selection bias was possible. Second, the cut-off values for each liver volume assessment method corresponded to the maximum sensitivity and specificity in predicting PHLF B/C in the ROC curve analysis, which may not apply to other centers. Third, the possibility of measurement bias cannot be ruled out because of the different instruments used to measure liver volume depending on the study period. Fourth, in cases of PVE and PBD for cholangiocarcinoma, the distribution of liver function is heterogeneous, and function of the preserved liver should normally be assessed. However, the main purpose of the present study was to assess liver volume; therefore, the heterogeneity of liver function was not considered. Fifth, although this study was specific to liver volume, the ability of the FLR to maintain the body’s metabolic demands after hepatectomy is also dependent on functional reserve and reperformance, and the liver regenerative capacity is influenced by the presence of underlying diseases, particularly cirrhosis [27]. Finally, while high BMI itself promotes liver regeneration [28], and tends to be a protective factor against PHLF, [27] it is a factor that increases the risk of serious complications [29], and low BMI has been reported to be a risk factor for coagulopathy [30]. Therefore, prospective studies involving larger patient cohorts are warranted. Considering the above, the next step would be to pursue the possibility of constructing a model that can more accurately predict PHLF by integrating liver function tests such as the ALBI score, 99mTc-GSA scintigraphy, or MRI-based function with the liver volume parameters investigated in this study. Despite these drawbacks, we believe that our results will interest hepatobiliary surgeons, as no previous report has described an optimal liver volume assessment method based on body mass.

In conclusion, any FLRV evaluation method can predict PHLF accurately in patients with standard body mass. However, different evaluation methods should be used depending on the body mass of patients deviating from the standard body mass. FLRV/BW and FLRV/TLV for lean patients and obese patients, respectively, were more useful than other evaluation methods.

## Supplementary Information

Below is the link to the electronic supplementary material.

Supplementary file1 (DOCX 229 KB)
