# Supplementary material for: Appropriate methods of evaluating future liver remnant volume to predict postoperative liver failure after major hepatectomy based on the body mass of patients with normal hepatic reserve
Source: Surg Today. 2025 Mar 27;55(9):1284–94. doi: 10.1007/s00595-025-03030-0 (PMC12380875; doi:10.1007/s00595-025-03030-0)
Supplement: Supplementary file 1 — Supplementary file1 (DOCX 229 KB) [file 595_2025_3030_MOESM1_ESM.docx]

**Online Resource 1.** Correlation between TLV and BMI in all the patients

The correlation between BMI and TLV calculated using CT in all the patients is illustrated. Here, r is the correlation coefficient.

BMI, body mass index; TLV, total liver volume; CT, computed tomography.


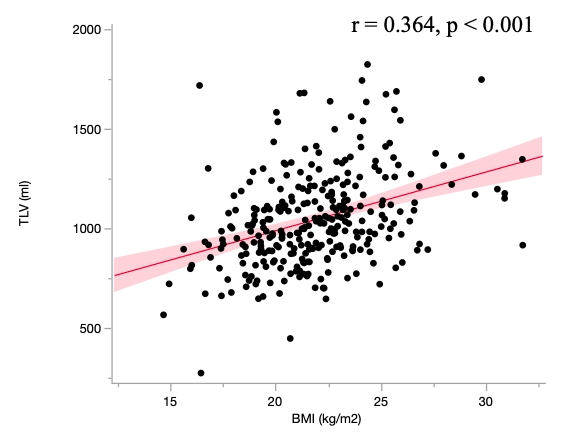


**Online Resource 2**. Cut-off values for each parameter and sensitivity and specificity when using those cut-off values in the subgroups

| parameters | **Standard body mass with 19.8 ≤ BMI ≤ 23.4 (n = 155)**  **Cut-off value 　　　　　　　　　　(sensitivity, specificity)** | **0bese cases with BMI > 23.4 　 (n = 76)**  **Cut-off value 　　　　　　　　　　　(sensitivity, specificity)** | **Lean cases with BMI < 19.8 　(n = 76)**  **Cut-off value 　　　　　　　　　　(sensitivity, specificity)** |
| --- | --- | --- | --- |
| FLRV (ml) | 455 (0.742, 0.762) | 506 (0.712, 0.800) | 419 (0.619, 0.727) |
| FLRV/TLV (%) | 40 (0.850, 0.455) | 40 (0.894, 0.500) | 40 (0.831, 0.455) |
| FLRV/SLV (%) | 42 (0.734, 0.762) | 40 (0.803, 0.700) | 45 (0.619, 0.727) |
| FLRV/BW (%) | 0.7 (0.930, 0.429) | 0.7 (0.833, 0.600) | 0.9 (0.698, 0.546) |

The cut-off values for each liver volume assessment were calculated mechanically as the value at which the Youden index calculated from the receiver operating characteristic (ROC) analysis was maximized.

FLRV, future liver remnant volume; TLV, total liver volume; SLV, standard liver volume; BW, body weight; BMI, body mass index.

**Online Resource 3**. Comparison of DM prevalence, serum AST and ALT levels between subgroups

DM, diabetes mellitus; AST, aspartate aminotransferase; ALT, alanine aminotransferase.

(a)

(b)

(c)

**Online Resource 4**. Area under the curve of each parameter on remnant liver volume for predicting grade B or C PHLF stratified by age

(a), (b): Obese group; (c), (d): Standard group; (e), (f): Lean grroup

(a)

| Parameters | Obese group  (n = 76) | Grade B or C PHLF  Yes (n = 10) No (n = 66) | | | | P |
| --- | --- | --- | --- | --- | --- | --- |
| Age, years* | 70 (37–84) | 73 | (48–80) | 70 | (37–84) | 0.853 |
| Age |  |  |  |  |  | 0.615 |
| < 70 | 36 (47%) | 4 | (11%) | 32 | (89%) |  |
| ≥ 70 | 40 (53%) | 6 | (15%) | 34 | (85%) |  |

(b)

| Parameters | **Age** < **70 (n = 36)**  **AUC (95% CI) P** | | **Age** ≥ **70 (n = 40)**  **AUC (95% CI) P** | |
| --- | --- | --- | --- | --- |
| FLRV | 0.680 (0.296, 0.914) | 0.255 | 0.686 (0.382, 0.886) | 0.228 |
| FLRV/TLV | 0.656 (0.280, 0.904) | 0.443 | 0.696 (0.378, 0.896) | 0.132 |
| FLRV/SLV | 0.695 (0.343, 0.909) | 0.196 | 0.710 (0.380, 0.908) | 0.191 |
| FLRV/BW | 0.703 (0.380, 0.902) | 0.170 | 0.711 (0.374, 0.910) | 0.166 |

(c)

| Parameters | Standard group  (n = 155) | Grade B or C PHLF  Yes (n = 22) No (n = 133) | | | | P |
| --- | --- | --- | --- | --- | --- | --- |
| Age, years* | 68 (27–88) | 68 | (56–81) | 68 | (27–88) | 0.735 |
| Age |  |  |  |  |  | 0.972 |
| < 70 | 84 (54%) | 12 | (14%) | 72 | (86%) |  |
| ≥ 70 | 71 (46%) | 10 | (14%) | 61 | (88%) |  |

(d)

| Parameters | **Age** < **70 (n = 84)**  **AUC (95% CI) P** | | **Age** ≥ **70 (n = 71)**  **AUC (95% CI) P** | |
| --- | --- | --- | --- | --- |
| FLRV | 0.603 (0.369, 0.798) | 0.574 | 0.850 (0.732, 0.922) | < 0.001 |
| FLRV/TLV | 0.626 (0.427, 0.790) | 0.219 | 0.882 (0.760, 0.946) | < 0.001 |
| FLRV/SLV | 0.598 (0.360, 0.797) | 0.600 | 0.865 (0.744, 0.934) | < 0.001 |
| FLRV/BW | 0.595 (0.360, 0.794) | 0.551 | 0.870 (0.751, 0.937) | < 0.001 |

(e)

| Parameters | Lean group  (n = 76) | Grade B or C PHLF  Yes (n = 11) No (n = 65) | | | | P |
| --- | --- | --- | --- | --- | --- | --- |
| Age, years* | 70 (1–85) | 72 | (57–83) | 70 | (1–85) | 0.120 |
| Age |  |  |  |  |  | 0.168 |
| < 70 | 35 (46%) | 3 | (9%) | 32 | (91%) |  |
| ≥ 70 | 41 (54%) | 8 | (20%) | 33 | (80%) |  |

(f)

| Parameters | **Age** < **70 (n = 35)**  **AUC (95% CI) P** | | **Age** ≥ **70 (n = 41)**  **AUC (95% CI) P** | |
| --- | --- | --- | --- | --- |
| FLRV | 0.473 (0.218, 0.743) | 0.838 | 0.650 (0.406, 0.835) | 0.290 |
| FLRV/TLV | 0.490 (0.300, 0.682) | 0.925 | 0.803 (0.594, 0.919) | 0.005 |
| FLRV/SLV | 0.495 (0.258, 0.734) | 0.988 | 0.664 (0.419, 0.844) | 0.241 |
| FLRV/BW | 0.559 (0.361, 0.740) | 0.800 | 0.660 (0.415, 0.842) | 0.290 |

*Values are median (range).

Values in parentheses are 95% confidence intervals (CIs). The area under the curve (AUC) for each parameter was calculated from receiver operating characteristic (ROC) curve analyses for predicting grade B or C post-hepatectomy liver failure.

PHLF, post-hepatectomy liver failure; FLRV, future liver remnant volume; TLV, total liver volume; SLV, standard liver volume; BW, body weight.
